# *Terminalia arjuna*, a Cardioprotective Herbal Medicine–Relevancy in the Modern Era of Pharmaceuticals and Green Nanomedicine—A Review

**DOI:** 10.3390/ph16010126

**Published:** 2023-01-13

**Authors:** Purnimajayasree Ramesh, Arunkumar Palaniappan

**Affiliations:** 1 School of Biosciences and Technology, Vellore Institute of Technology, Vellore 632-014, India; 2 Centre for Biomaterials, Cellular and Molecular Theranostics, Vellore Institute of Technology, Vellore 632-014, India

**Keywords:** plants, *Terminalia arjuna*, bioavailability, nanomedicine, phytochemicals, extracts, green synthesis, herbal medicine

## Abstract

Herbal medicines were the main source of therapeutic agents in the ancestral era. *Terminalia arjuna* (TA) is one such medicinal plant widely known for its several medicinal properties, especially its cardiovascular properties. They have several phytochemicals, such as flavonoids, polyphenols, triterpenoids, tannins, glycosides, and several minerals, proteins, and others that are responsible for the above-mentioned medicinal properties. In this review, we have first elaborated on the various processes and their parameters for the efficient extraction of relevant phytochemicals from TA extracts. Secondly, the mechanisms behind the various medicinal properties of TA extracts are explained. We have also highlighted the role of TA extracts on the green synthesis of metallic nanoparticles, especially silver and gold nanoparticles, with an elucidation on the mechanisms behind the synthesis of nanoparticles. Finally, TA extracts-based polymeric formulations are discussed with limitations and future perspectives. We believe that this review could help researchers understand the importance of a well-known cardioprotective medicinal plant, TA, and its biomedical properties, as well as their role in green nanotechnology and various formulations explored for encapsulating them. This review will help researchers design better and greener nanomedicines as well as better formulations to improve the stability and bioavailability of TA extracts.

## 1. Introduction

In the bygone period, people relied mainly on the plant kingdom for their medicines to cure various health disorders. As per World Health Organization (WHO) statistics, 80% of the world’s population still consumes traditional herbal supplements and medicines [1,2]. Unlike synthetic drugs, medicinal herbs are easily available, especially in low-income countries, and are also generally considered to be non-toxic with fewer side effects. Thus, they are often the preferred treatment option, especially in underdeveloped or other developing countries [2]. With the advent of vertical farming and advancements in biotechnology, there is more scope for these medicinal herb-based systems to get popular in developed countries as well as in the coming years [3]. These medicinal herbs are currently being utilized in pharmaceuticals [4], food preparations [5], nutraceuticals [6], folk medicines [7], and many others due to the biological properties of the bioactive molecules in the plants [8,9].

**Figure 1:**
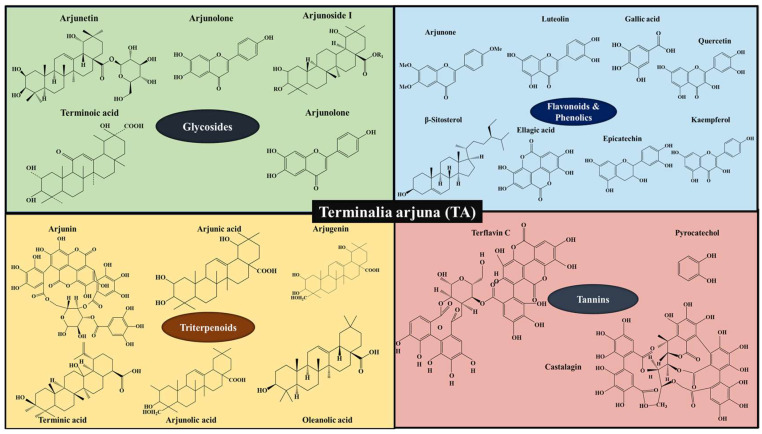
Major phytochemicals of TA used in biomedical applications.

*Terminalia arjuna* (TA) is one such folklore medicinal herb, and it belongs to the Combretaceae family. The plant is mainly found in the Indian subcontinent. There are nearly 24 species in India, and it is a deciduous and evergreen tree that grows up to 20–30 m above the ground level. The different parts of the plant, such as the fruit [10], bark [1], leaf [11], seed [12], and root [13] are found to possess different medicinal properties. Among them, barks are found to have rich medicinal value. It is one of the most commonly used plants in Siddha, Ayurveda, and Unani systems of treatment. In India, TA is recognized by different local names such as Arjuna/Arjun (Hindi), Marudhu (Tamil and Malayalam), Yella maddi (Telugu), Arjhan (Bengali), Sadaru (Marathi), Sadad (Gujarati), and Neer matti (Kannada) [9,14,15]. The phytochemicals extracted from TA (Figure 1) are found to have rich antioxidant properties in addition to several other bioactive properties, such as antioxidant [16], anti-inflammatory [[17], cardio-protective [18,19], anti-atherosclerotic [20], and anti-tumor [21]. TA exhibit various pharmaceutical properties when treating various clinical disorders, such as heart failure, ischemia, cardiomyopathy, atherosclerosis, myocardium necrosis, tumor, viral diseases, ulcer and many others [1].This review briefly explains the underlying mechanisms of the above-mentioned properties and their role in various pharmaceutical applications. In addition, this review also focuses on the various types of polymeric formulations to enhance the solubility, bioavailability, and stability of phytochemicals from TA extracts.

The metallic nanomaterials are found to have increased applications in healthcare and environmental applications [22,23]. They are typically synthesized using chemical methods, wherein potent reducing agents are used [24]. Though potent, they are associated with various toxicity issues [24]. Thus, green methods of nanomaterial synthesis are becoming popular [25]. Plant extract-based nanomaterial syntheses are one such method that is found to have low toxicity and more potent medicinal properties with respect to the plant extracts being used. TA-based extracts have been reported in the literature for the syntheses of a wide gamut of nanomaterials. In this review, we also highlight various studies on the green synthesis of metallic nanomaterials using TA-based extracts, the mechanisms behind the syntheses, and their biomedical applications.

## 2. Extraction of Phytochemicals from TA

Extraction is the initial stage to obtain the bioactive molecules from the desired part of the plant. Medicinal plant extraction has a sequence of steps that begins with the collection and authentication of the plant, then drying, and ends with the extraction, fractionation, and isolation of bioactive molecules. The efficacy of extraction solely depends on the solvents and the process used. The extraction process depends on the solvent’s interaction with the sample to dissolve and diffuse out the desired phytochemicals of interest. The efficiency of the extraction process is mainly based on the particle size of the plant parts, the duration of the extraction process, the temperature, and the solvent to plant mass ratio [26].

The conventional methods of extraction include maceration [27], infusion [28], percolation [29], decoction [30], hot continuous extraction (Soxhlet extraction) [31], and hydro-distillation [32]. The main limitations of traditional extraction methods include their excessive solvent consumption, time-consuming process, low productivity, lack of effectiveness, and inapplicability to all types of components. Modern extraction methods have many advantages, such as being less time-consuming, having high reproducibility, easy automation, and a low utilization of toxic solvents compared to the conventional methods. These methods include supercritical fluid extraction [33], microwave-assisted extraction [34], ultrasound-assisted extraction [35], enzymatic hydrolysis [36], and others [37].

The extraction process of bioactive compounds involves the separation and identification of phytoconstituents, followed by the characterization of extracted plant molecules. Different extraction techniques have different objectives, which range from the selection of the target molecule to the enhancement of selectivity and sensitivity, the conversion of the biomolecules to a suitable form, and above all, to provide a robust and reproducible method to prepare the sample [26].

### 2.1. Pre-Extraction Process for a TA Plant

The pre-extraction process involves the preparation of the plant materials for the improvement of the efficacy of the extraction process. Moreover, these processes assist in the preservation of phytochemicals prior to extraction.

#### 2.1.1. Drying of TA Plant Materials

Drying is the most important and predominant part of the pre-extraction process [38]. The drying of plant material is usually performed to protect the sample from mold or microbial infestation, tissue deterioration, and phytochemical alterations caused by enzymes or microbes [38]. The drying impacts the quality of the plant material and is usually based on four factors: temperature, humidity, airflow, and the cleanliness of the air. In order to increase the shelf life of the plant material, the ideal moisture content of the plant is reported to be below 12% after drying [38]. The plant materials are typically dried at a temperature below 30 °C, away from the sunlight, with proper air circulation to avoid the degradation of phytoconstituents and to protect the sample from heat and moisture. Likewise, fresh samples are used within 3 h of the collection because the plant materials are susceptible to decomposition if left for longer periods. The field extraction of phytoconstituents with solvents is also reported, but the main limitation of the field extraction is that it will inactivate enzymes in the plant, so alternative methods, such as freezing and preserving in alcohols, are highly preferred [37,39].

#### 2.1.2. Grinding and Powdering of TA Plant Materials

Lowering the particle size increases the surface area for the interaction between the sample and the extraction solvent. To obtain an efficient extraction of the desired phytoconstituents, the solvent must make good contact with the target molecules. Furthermore, the appropriate size of the particles is reported to be smaller than 0.5 mm [37]. The optimum particle size is preferred because it increases the surface area, which will enhance the bulk transfer of active phytochemicals from the plant to the solvent. Hence, the preferred size of particles interact with the solvent and release the phytochemicals, while the larger particles sediment at the bottom and turn slimy during extraction [40]. Traditionally, a mortar and pestle are used to grind the sample. However, in recent times, modern techniques such as the use of a planetary ball miller have been used to grind the sample [37].

#### 2.1.3. Effect of Solvents and the Phytochemicals Polarity on the Extraction Process Efficiency

Solvents are the vital factor in the phytochemical extraction process. They are selected based on the polarity of the molecule and can be classified as polar or non-polar solvents. The polar solvents include water, methanol, ethanol, and others, while the non-polar solvents include hexane, dichloromethane, and others. In the solvent category, water has the highest polarity while hexane has lowest polarity [41]. Table 1 summaries the phytochemicals derived from various parts of TA plant.

TA is a rich source of bioactive molecules and can thus be extracted by both conventional and modern methods. The therapeutic efficacy is based on the extraction method and choice of the solvent. The yield of phytoconstituents is based on the menstruum used. The polar and non-polar solvents are used in the extraction process in the order of increasing polarity [41,65].

The highly polar polyphenols that are extracted using water are the key bioactive molecules of TA. Hence, the aqueous extract of TA has 44% by weight of polyphenols, which are polymeric in nature. The non-polar polyphenols, catechins, and flavonols are isolated by sequential extraction with the use of methyl isobutyl ketone and ethyl acetate. Likewise, *n*-butanol extracts the mid-polar polyphenols. The catechins, gallocatechins, and ellagic acid are extracted using methanol [42,66].

Triterpenoids are usually non-polar compounds, but their polarity is increased when they react with oxygenated substituents, such as hydroxyl and carboxyl groups (methanol-ethyl acetate) [67]. So far, to extract triterpenes, solvents such as chloroform, dichloromethane, methanol, etc. are utilized [68,69,70].

In TA, tannins belong to the polyphenolic group, which are complex secondary metabolites and are extracted using both polar and non-polar solvents. For instance, the difference in the percentage of tannins obtained from the aqueous and alcoholic extracts of the bark was found to be 16% and 12%, respectively (26,37,39,40).

Thus, obtained extracts are used to treat various biomedical problems, such as heart failure, ischemia, cardiomyopathy, atherosclerosis, myocardium necrosis, blood diseases, anaemia, venereal, fractures, ulcer, and viral disease. The plant is renowned for its cardio-protective properties. This review aims to describe the mechanism of action of the anti-inflammatory, antioxidant, cardioprotective, anti-atherosclerotic, and anti-tumor properties of TA.

## 3. Mechanism of Action

### 3.1. Antioxidant

Free radicals are molecular species that donate or accept electrons from other molecules and thus act as oxidants or reductants. These free radicals are unstable and highly reactive (hydroxyl radical, superoxide anion radical, hydrogen peroxide, oxygen singlet, nitric oxide radical, hypochlorite, and peroxynitrite radical), resulting in the damage to cells as well as the disruption of homeostasis. In the human body, free radicals are generated as a result of a few essential metabolic processes, such as exercise and ischemia/reperfusion injury, or due to exposure to harmful radiation sources (such as x-rays, UV, and others) and toxic chemicals (such as cigarette smoke or ozone fumes, to name a few) [71].

Antioxidants are the chemicals that counteract and stabilize the excessive production of free radicals and enhance the immune response. These are generally produced inside the body, or they can be given as a supplement to prevent diseases [71]. Natural herbs are considered to be a rich source of these antioxidants. The phytochemical constituents that are mainly responsible for the antioxidant property are the phenolic compounds [72].

**Figure 2:**
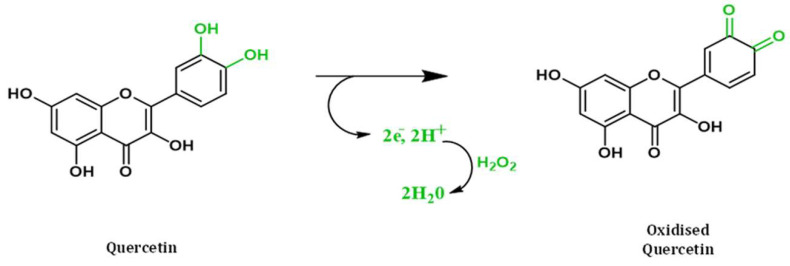
Mechanism of hydrogen radical scavenging activity of quercetin (flavonoid) present in TA extracts were reproduced with permission [73].

The major flavonoid and phenolic compounds in the leaves of TA include gallic acid, apigenin, luteolin, quercetin, epicatechin, ellagic acid, and 1-*O*-β-galloyl glucose, and they are identified as the sources for TAs antioxidant activity [47]. Plant biomolecules act as electron donors, reductants, singlet oxygen quenchers, and chelate metal ions to exhibit their antioxidant property [43]. The mechanism behind phenolic compounds’ exhibiting the antioxidant property is that phenolic compounds in plants possess one or more aromatic groups along with one or more hydroxyl groups. The antioxidant potential of the phenolic compounds is due to the conjugation and side chains of the aromatic rings with the generated free hydroxyl radicals. The another antioxidant mechanism in which flavonoids exerts its property is through scavenging the hydrogen peroxide radicals [16]. For instance, hydrogen peroxide is mainly formed by superoxide dismutase within the cells. The flavonoids donate electrons or protons to convert the produced hydrogen peroxide into water. The equation below in Figure 2 represents the possible mechanism of hydrogen peroxide radical scavenging activity of the quercetin (flavonoid) molecule [73].

The role of solvents plays a key role in extracting the right phytochemicals with antioxidant properties. For instance, in the case of phytochemical extraction from TA leaves, the aqueous extraction was found to have the highest free radical scavenging properties when compared to other extraction processes involving other solvents, such as benzene, acetone, petroleum ether, hexane, and chloroform [74,75]. The chloroform extracts non-polar compounds, such as triterpenes and lipids, and the n-butanol extracts mid-polar compounds, such as oxidized catechins and flavanol glycosides, while highly polar compounds, such as tannins and polyphenols, reside in water [42].

Likewise, the methanolic extract of TA bark also exhibits high antioxidant potential, which is mainly due to its flavonoid content [43]. Methanol and ethanol are the solvents generally preferred to extract molecules with antioxidant properties. However, ethanol is highly preferred due to its low levels of toxicity. The polarity and increased solubility of many antioxidant molecules in methanol make it an ideal solvent chemically, although its toxicity limits its applications. With an increase in the concentration of the extract, there is an increase in the reducing power, which is a key indicator in determining the antioxidant activity [72]. Scavenging of hydrogen peroxide (H_2_O_2_) is important because it gives hydroxy radicals to cells, which are toxic to cells. It can be neutralized by elevating the dosage level of phytochemical extracts. The phenolic compounds will scavenge the hydrogen peroxide by donating one electron and neutralizing it, leading to the formation of water. The methanolic extract exhibits the highest antioxidant activity, followed by ethanolic extracts, while the lowest activity was found in the n-hexane solvent [64].

### 3.2. Anti-Inflammatory

Inflammation is usually caused by infection, xenobiotics, and a weak immune response. The inflammatory response involves the recruitment of macrophages and neutrophils in the initial phase, which secrete different mediators to cause acute inflammation. Nitric oxide is a key mediator that induces inflammation through the expression of inducible (iNOS). Plant flavonoids inhibit nitric oxide (NO) production and downregulate the expression of iNOS (nitric oxide synthase). Flavonoids also inhibit the biosynthesis of prostaglandins by inhibiting the enzymes cyclo-oxygenase and COX-1 and 2 at the molecular level. The pro-inflammatory cytokines are also inhibited by these flavonoids [76].

**Figure 3:**
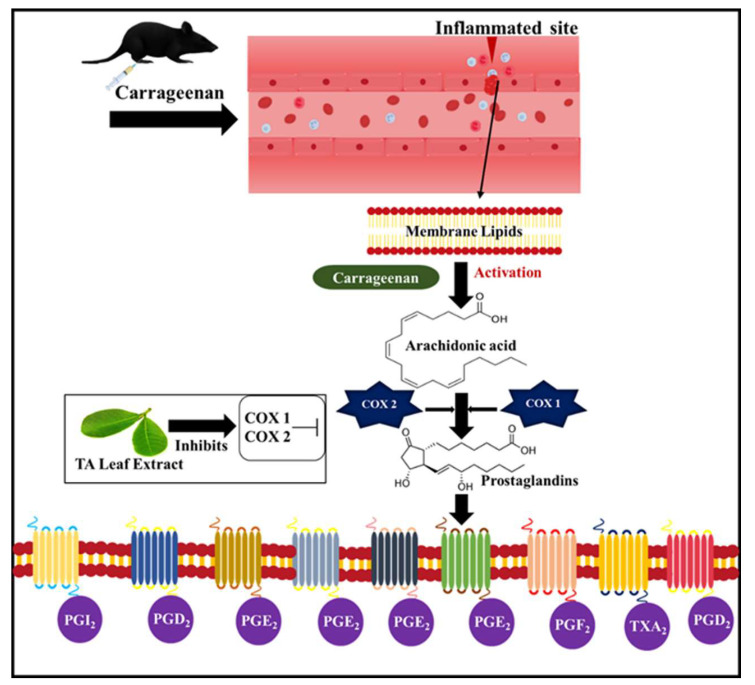
Anti-inflammatory mechanism of action of TA extract on Carrageenan-induced paw edema.

The anti-inflammatory study on the TA leaf was reported for its methanolic extract. The methanolic extract of the leaf efficiently reduced the carrageenan-induced paw edema. The possible mechanism reported was the inhibition of COX enzymes, which leads to the inhibition of prostaglandin synthesis, as depicted in Figure 3 [17].

The commercially available TA capsules (containing TA bark extract) were also studied for their anti-inflammatory activity in paw edema models developed using formalin and carrageenan. It was reported that the plausible anti-inflammatory mechanism could be any of the following: (1) blocking the biosynthesis of proinflammatory mediators (COX II); (2) inhibiting the release of mediators (e.g., histamine, a potent inflammatory mediator); (3) blocking the mediator-receptor (e.g., leukotriene receptor, an inflammatory mediator) interactions; and (4) immune stimulation (e.g., myeloid cell maturation). It was also reported that the TA extract increased the amount of antioxidant enzymes (superoxide dismutase and catalase) as well as reduced the glutathione content, which as a whole results in the enhancement of anti-inflammatory activity [77,78]. For instance, during inflammation, the neutrophils are recruited at the site to initiate the release of ROS and chemokines. The release of superoxide anions activated the endothelial cells, generated chemotactic mediators (leukotriene B4), and upregulated adhesion molecules that increase neutrophil infiltration. The neutrophils under oxidative stress generate reactive oxygen species, where the antioxidant enzyme SOD acts as an endogenous cellular defense system and degrades the superoxide into oxygen and hydrogen peroxide, where the generation of hydrogen peroxide activates the caspases three, eight, and nine, to induce neutrophil apoptosis [79].

### 3.3. Cardio Protective Property

TA has always been considered a cardio tonic plant since the ancient era and is currently gaining importance in the treatment of various cardiac disorders. The alcoholic extract of TA includes lactones, flavonoids, phytosterol, phenolic compounds, glycosides, and tannins that have excellent antioxidant, antihyperlipidemic, and cardio protective properties [80]. The cardiovascular disease is caused due to many factors, such as hyperlipidaemia, hyperglycaemia, inflammatory response, coagulation factors, increased platelet activation, smoking, and oxidative stress [81].

**Figure 4:**
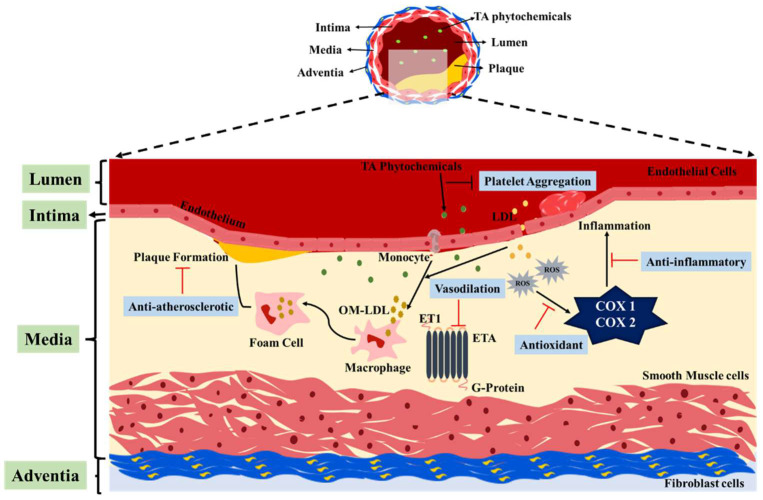
Cardioprotective mechanism of action of TA.

The cardio-protection is studied using the gemmomodified (plants at growing stage) extract and the methanolic extract of the TA bark. The HPLC analysis of the extracts revealed that bark has a higher concentration of flavonols (quercetin, myricetin, and kaempferol), flavanole (catechin), and phenolic acid (gallic acid, *P. coumaric*, and ferulic acid). The mechanisms behind TAs cardiac protection properties are as follows: 1. Flavonoids present in them reverse endothelial dysfunction and reduce the arterial pressure by increasing the vasorelaxation process. 2. The flavonoids also scavenge the free radical species through scavenger receptors and help in the uptake and elimination of oxidized modified low-density lipoprotein (OM-LDL)(OM-LDL is a chemically modified LDL that results in endothelial injury to initiate atherosclerotic plaque formation [82]). 3. Regulation of platelet activity and by inhibition of platelet aggregation as depicted in Figure 4. Both the bark extract as well as the gemmomodified extract have potent properties to cure induced myocardial infarction [61].

The aqueous ethanolic extract of bark has been studied for various factors that cause chronic heart failure. For instance, lipids play a vital role in cardiovascular diseases since the alteration in lipid metabolism manipulates cardiac functions by affecting the structure, stability, and composition of the cell membrane, which in turn leads to cell death followed by ischemia. This extract helps by reducing the LDL and increasing the HDL, which proves the lipid-lowering properties of the plant extract. In other instances, the cell membrane of the myocardial cells gets damaged, which results in the leakage of the enzyme, creatine kinase. The treatment with aqueous ethanolic extract maintains the membrane’s integrity by increasing the level of creatine kinase and thereby stopping the leakage of enzymes [83]. Another study revealed that the cardio-protection properties of TA are due to the following reasons: (a) through the antioxidant properties of tannins and (b) through activating the physiological antioxidants inside the body by the oleanane triterpenoids [84].

### 3.4. Anti–Atherosclerotic

Atherosclerosis is an inflammatory vascular disease caused by the deposition of cholesterol in the arterial wall. Atherosclerosis is one of the major causes of cardiovascular diseases. The key factors responsible for plaque formation are the stimulation and differentiation of monocytes in the blood stream, followed by the low-density lipoprotein uptake by macrophages to form the foam cells [85]. The different ethanolic derivatives of TA demonstrate the presence of phenolics, tannins, triterpenoids, saponins, anthraquinone glycosides, alkaloids, and flavonoids and confirm the anti-hyperlipidemic activity through its anion exchange property. The main mechanism behind the anion exchange property is that the anions of phytochemicals in the plant bind with the bile acid anions in the intestine and convert cholesterol into bile acid, which is then excreted in stool and leads to a decrease in the serum LDL cholesterol level [86]. Arjunic acid and Arjunoglycoside (I, II, III and IV) undergo a drug-metabolizing cascade, produce some active molecules, and are responsible for lipid-lowering activity [20]. Tannins also are found to be helpful in depleting the lipid activity [87].

**Figure 5:**
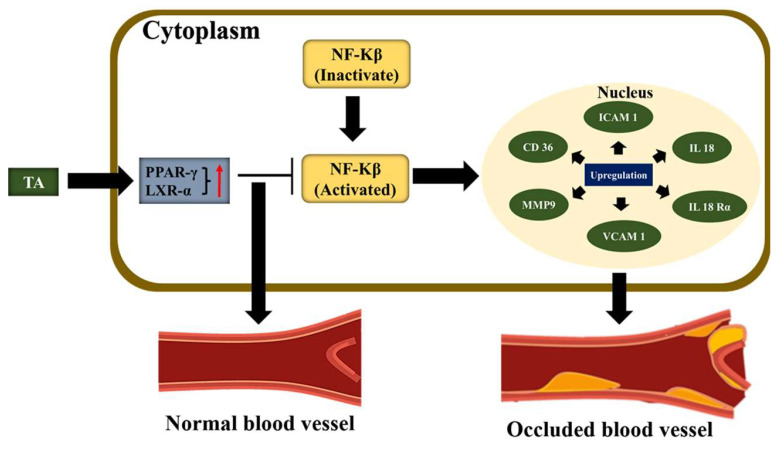
Schematic illustration of plausible mechanism behind the anti-atherosclerotic activity of TA extracts. The red arrow in the figure indicates the upregulation of nuclear receptors (PPAR-γ and LXR-α).

There is another mechanism reported that uses the aqueous bark extract of TA to treat atherosclerosis. The medicinal herbs are rich sources of ligands for nuclear receptors, such as the peroxisome proliferator-activated receptor α (PPARα) and the peroxisome proliferator-activated receptor γ (PPAR γ). Figure 5 provides a schematic representation of the mechanism of TA to inhibit NF-κβ and thus prevent plaque progression. Treatment with this extract activates the PPAR-γ by inhibiting the NF-κβ, which reduces the action of Interleukin 18 (IL 18) and also inhibiting the expression of other cytokines, chemokines, and adhesion molecules, and finally stops the plaque progression. Additionally, the TA extract also soothes the inflammatory cascade through pleiotropic activation of PPAR–γ and LXR (liver X receptors)–α as PPAR–γ upregulates the LXR–α and promotes cholesterol efflux and it also regulates the scavenger receptor (CD36) activation which helps in uptake of oxidized LDL in foam formation. The reduced expression of matrix metallopeptidase 9 (MMP 9) is also another possible mechanism to reduce atherosclerotic plaque formation [88].

### 3.5. Anticancer

Cancer is a life-threatening disease that often results in death. The current treatment strategies include primarily surgery, radiotherapy, and chemotherapy, with few adjuvant therapies. The above-mentioned treatment strategies are very harsh on patients, resulting in pain and serious side-effects, such as the loss of hair and severe pain, to name a few. In order to reduce the side-effects of the existing cancer treatment strategies, herbal medicines are currently much preferred. These phytoconstituents are reported to have excellent properties, such as (a) methyltransferase inhibition, (b) DNA damage prevention or antioxidant properties, (c) histone deacetylases inhibition, and (d) mitotic disruption that prevents cancer development.

**Figure 6:**
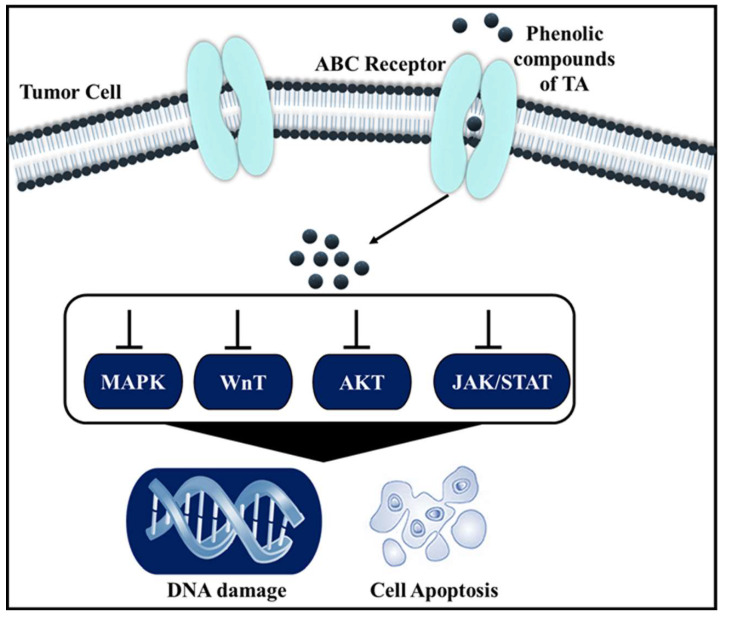
Schematic representation of the mechanisms behind the anticancer activity of TA extracts.

The secondary metabolites, such as polyphenols, brassinosteroids, and taxol, are found to possess anti-cancer activity [89]. In TA, the high content of tannin and polyphenols is mainly responsible for the anticancer activity [90]. Besides the phytoconstituents, the taxol is also produced from Pestalotiopsis terminaliae, an endophytic fungus found in the leaves of TA. Taxol is a highly preferred drug for the treatment of human malignant cancers. In cancer cells, microtubule formation plays a major role in mitosis, motility, cell shape, and cellular response. The microtubule is comprised of α and β tubulin heterodimers which helps in the assembly of microtubule network through polymerization. The α and β tubulin are the globular proteins with three functional domains (N-terminal, C-terminal, and an intermediate domain). Taxol binds with the β-tubulin on the intermediate domain to suppress the spindle formation and deploy the mitotic division, thereby arresting the cell division of the malignant cells [90,91,92]. TA extracts are also tested in colon and liver cancer cell lines for their anti-cancer activity. It was found that the bioactive molecules in the TA leaf extract destroy the cancer cells either by activating apoptosis or through the inhibition of the growth regulators [93]. The anticancer activity of TA bark is reported to be due to the presence of phenolic compounds that interact with target DNA through signaling pathways, as depicted in Figure 6, and block the sites involved in the electrophilic attack by reactive carcinogenic moieties [21].

## 4. Green Synthesis of Nanoparticles Using TA Extracts

Nanotechnology is an emerging and interesting multidisciplinary field of science that deals with particles/materials of a dimension of 1–100 nm [94]. Metallic nanoparticles have many unique properties, such as a high surface area, high surface energy, and quantum confinement when compared to their bulk counterparts [95]. There are three most commonly used methods of metal nanoparticle synthesis: physical (ball milling [96], plasma arcing [97], spray pyrolysis [98], sputter deposition [99], etc.), chemical (electrodeposition [100], sol-gel process [101], co-precipitation method [102], etc.), and biological methods (using plants, algae, and microorganisms). Among the three different methods of nanoparticle synthesis, biological methods are gaining popularity due to their sustainable and green approach, mostly with one-step methods of bio-reduction with less energy consumption and an eco-friendly approach [103]. On the other hand, the physical and chemical methods suffer from some issues, such as being highly energy intensive, utilizing radiation, and requiring a high solvent consumption, which are harmful for human health [95].

In biological methods, various sources such as algae [104], microorganisms [105], and plants [10] are considered efficient and eco-friendly green nano-factories that are capable of converting metal ions to metal nanoparticles (Ag, Au, Cu, Pd, Fe, and Zn) that can be utilized in various biological applications [106]. In microbes, the reductase enzyme, microbial proteins, metal resistance genes, and the reduction of co-factors play an important role in the synthesis of nanoparticles. Organisms such as bacteria, yeast, and fungi are the nano-factories that act as reducing and capping agents. Whereas, in phyto-nanotechnology (plant-based nanoparticle synthesis), organic acids, vitamins, proteins, and secondary metabolites such as alkaloids, flavonoids, terpenoids, polysaccharides, and heterocyclic compounds support the synthesis of nanoparticles. The different parts of a plant, such as the bark, root, fruit, oil, leaf, and flower, are all utilized in biological synthesis [94,107]. The size, shape, and composition of nanoparticles depend on the parts and phytoconstituents of the plants [108,109].

**Figure 7:**
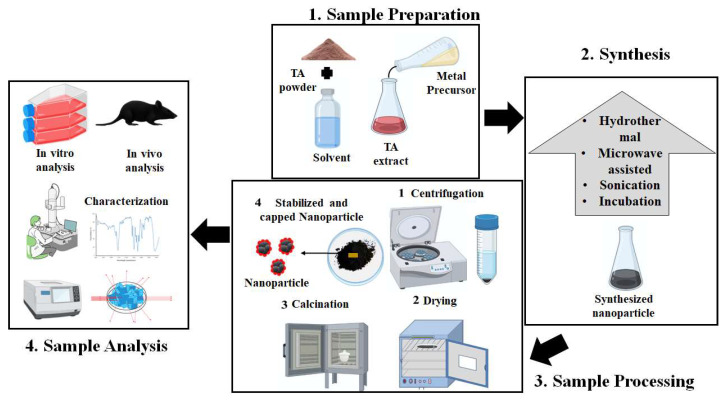
Schematic representation of the various steps involved in the green synthesis of metallic nanoparticles using TA extract.

TA is used in the synthesis of different metal nanoparticles. Figure 7 depicts the schematic overview of the green synthesis of metallic nanoparticles using TA. This review explains the synthesis of various types of nanoparticles from TA extracts and their biomedical applications. Various parts of TA are used in the synthesis of metallic nanoparticle. Till dates, metallic nanoparticles, such as gold, silver, copper, iron, and palladium, are synthesized using TA extracts. The most common methods employed in the nanoparticles’ syntheses are microwave irradiation [46], boiling [110], hydrothermal [111], sonication [111], stirring [112], wet chemical [111], and shaking [113], which each take place under different conditions.

### 4.1. Silver Nanoparticles

The synthesis of silver nanoparticles using a plant extract requires vital elements such as a silver metal ion solution and a capping agent. The polyphenols and protein molecules in TA extracts act as capping and reducing agents. The peptides degrade into smaller peptides, whose carboxylate groups help in the stabilization of nanoparticles by forming the corona layer on their surface [114]. In the green synthesis for the reduction of silver ions, a precursor silver nitrate is used along with the plant extract. The size of the nanoparticle can be controlled by environmental factors such as pH, temperature, precursor concentration, extraction time, and extract concentration. The size-controlled silver nanoparticles can be utilized for various biological applications [114].

**Figure 8:**
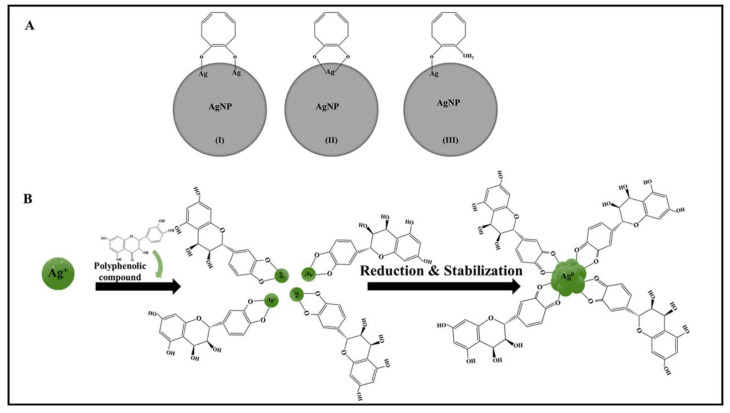
Schematic illustration of mechanism behind polyphenol in metallic nanoparticle synthesis, (**A**) catechol binding mechanism with silver nanoparticles, (**B**) silver nanoparticle synthesis were reproduced with permission [115].

The major bioactive molecules involved in the phyto-reduction process of silver nanoparticles are alkaloids, terpenoids, flavonoids, ketones, aldehydes, amides, tannins, quinines, and carboxylic acid [114]. Among all the bioactive molecules in TA, polyphenols are the key molecules that are used in the reduction of silver ions to form silver nanoparticles (Ag^+^ to Ag^0^). The metallic nanoparticles synthesized by the phenolic compounds have a high stability when compared with other reducing agents, such as citrate or sodium borohydride [106,115]. The natural phenols with hydroxyl and carbonyl groups have protonating and metal absorbing capabilities, while the catechol group of some phenolic compounds has only a metal-absorbing capability. The catechol functional group absorbs the metal surface through three different configurations, including a bidentate bridging bond, a bidentate chelating bond, and a monodentate-ester-like bond (Figure 8A) [46,106,115]. The phytoconstituents are the key molecules that help in mediating the synthesis reaction by performing the dual task of reducing and capping the agent. Further, it also acts as a stabilizer in the post-synthesis process, as illustrated in Figure 8B [116]. In the case of the flavonoids, the keto group helps in reducing silver to silver nanoparticles while converting flavonoids to an enol form [114].

### 4.2. Gold Nanoparticles

TA extract acts as a reducing agent for the synthesis of gold nanoparticles with the help of a precursor called chloroauric acid (HAuCl_4_). Molecules such as arjunetin, leucoanthocyanidins, and hydrolysable tannins are reported to be involved in the gold nanoparticle synthesis [117]. Furthermore, the green synthesis has a low cytotoxicity compared to chemical synthesis [118]. The main ingredient of TA that helps in the gold nanoparticle synthesis is polyphenols [119,120]. The polyphenolic compound can also be used for in situ stabilization of gold nanoparticles. The gold nanoparticles are synthesized and stabilized by the following reported mechanism: Au^3+^ is chelated by the adjacent hydroxyl group of the polyphenolic compound to form a five-membered chelate ring. Thus, the chelated o-dihydroxyl becomes oxidized to quinones and thus helps in the further reduction of Au^3+^ to Au^0^. The oxidized quinone stabilizes the gold nanoparticle and prevents aggregation [119]. Table 2 summarizes the metallic nanoparticles synthesized using TA extract and their role in the biomedical application.

## 5. Polymeric Formulation of TA Extracts

The phytochemicals extracted from TA extracts have several beneficial properties, as mentioned above; however, they suffer from limitations, such as low bioavailability, low stability, and low therapeutic efficacy. In order to overcome the above-said issues, a few polymeric formulations were explored [109,138]. There is one study in the literature where they have investigated the possibility of encapsulating the TA extract inside a few synthetic polymeric materials, such as Poly (lacto-co-glycolic acid) (PLGA), Polycarbonate (PC), Polypropylene (PP), Polyethylene (PE), Polytetrafluoroethylene (PTFE), and Chitosan [139]. However, there are no detailed investigations on the extract release kinetics and other pharmacokinetic studies. Further in-depth investigations are warranted. The below section summarizes a few studies relevant to the encapsulation and prolonged release studies of TA extracts using polymeric biomaterials.

### 5.1. Transdermal Delivery of TA Extracts

Transdermal delivery is one of the least invasive systems for delivering drugs directly to the blood stream by skipping the first pass metabolism in the liver and also increasing the bioavailability of the TA extract. A TA-extract-loaded chitosan ERL100 transdermal patch has been reported in the literature. Chitosan has good muco-adhesive properties and greater solubility in acidic aqueous solutions. The co-polymer Eudragit RL 100 (ERL 100) (containing acrylic acid and methacrylic acid esters as co-polymers with low quaternary ammonium groups) helps convert the polymer into salt to further enhance the permeability of the polymer. The polymeric patch was developed using the solvent evaporation technique. The designed patch was stable for three months and showed a controlled TA extract release for a prolonged time of up to 12 h with no allergic reactions in albino rats for 72 h [138].

There is another report on the emulgel-based transdermal delivery systems for TA extracts. The methanolic TA extract is emulsified using oil, surfactant, and co-surfactant and is fed into carbopol hydrogel. The in vitro and ex vivo release studies of the extract from the emulgel were conducted, and the maximum and prolonged release were found in 500 mg of the extract. The in vitro maximum release percentage was found to be 91.43% in 720 min and in ex vivo it was found to be 89.32% [140].

### 5.2. TA Bark Gum as a Biomaterial for Ophthalmic Application

Stimuli-responsive gels are an attractive material of choice for drug delivery in ophthalmic applications. pH-responsive gelling systems are an ideal candidate, as they can be transformed into gels upon a change in pH. In one study, gum from TA bark was combined with sodium alginate (SA) to fabricate an in situ gelling system for the delivery of moxifloxacin HCl (MOX-HCl). The system was found to be a clear solution before injection and was converted into a gel in the presence of tear solution. Furthermore, the gel was found to release the drug in a sustained and controlled manner for twelve hours with no signs of irritation or any other abnormalities such as inflammation or increased tear secretion. Thus, TA bark gum blended with SA in situ gel containing MOX-HCl was reported as an eye-drop replacement for extended precorneal retention, improved corneal permeability, and improved ocular bioavailability [141].

## 6. Conclusions and Future Perspectives

*Terminalia arjuna* is a medicinal plant that has several medicinal properties, such as anti-inflammatory, antioxidant, anti-ischemic, anti-atherosclerotic, antimicrobial, anti-cancer, anti-fertility, anti-mutagenic, etc. These properties are due to the presence of a variety of phytochemicals, such as flavonoids, polyphenols, triterpenoids, tannins, glycosides, and several others, in the extracts. The extraction of the desired phytochemicals depends on several factors, such as solvent properties, phytochemical properties, extraction methods, and many others, which are detailed in this review. The synthesis of metallic nanomaterials, especially gold and silver nanoparticles, using TA extracts is elaborated; however, more detailed physicochemical characterizations and mechanistic understandings are warranted. More in-depth in vitro and in vivo studies are needed to understand the advantages of these nanomaterials when compared to their chemically synthesized counterparts.

Although TA plant extracts have several unique and beneficial biomedical properties, as elaborated in this review, their potential is not yet utilized to the fullest. We expect more investigations on various types of formulations, such as nano/micro encapsulation technologies and scaffold- and hydrogel-based encapsulation technologies, to enhance the bioavailability and stability of the TA extracts. Moreover, there is also a need to further investigate detailed mechanisms of action using advanced omics studies to have a system-level view on the efficacy and toxicity of these extracts. Furthermore, there is a need for long-term toxicity studies on these extracts to be convincingly approved by regulatory authorities.

## Figures and Tables

**Table 1:** Summary of the phytochemicals derived from various parts of TA plant, their extraction methods, and their biomedical applications.

S. No	Part Used	Extraction Method	Solvent Used	Phytochemicals Derived	Application	Reference
1.	Bark	Refluxing	Water	Polyphenols	Polyphenol analysis	[42]
			Methanol	Catechins Gallocatechins Ellagic acid		
2.	Bark	Soxhlet	Methanol	Phytosterol Lactones Flavonoids Phenolic compounds Tannins Glycosides	Antimicrobial Antioxidant	[43]
3.	Bark-B Leaf-L Fruit–F	Shaking incubation	Hexane	Alkaloid–F, L Steroids–F	Antioxidant DNA nicking inhibition	[44]
			Ethyl acetate	Steroids–B, F Alkaloids-B Flavonoids-B Tannins and Phenolics–L		
			Chloroform	Steroids-F Alkaloids–F, L Tannins and Phenolics-L Saponins–L		
			Acetone	Tannins and phenolics Steroids Alkaloids absent only in leaf Flavonoids Saponins		
			Ethanol	Steroids Alkaloids Flavonoids Saponins Tannins and phenolics		
			Methanol	Steroids Alkaloids absent only in leaf Flavonoids Saponins Tannins and phenolic		
			Distilled water	Tannins and phenolic Steroids–B Alkaloids–L Flavonoids–B&F Saponins		
4.	Bark	Soxhlet	Methanol	Phenols Flavonoids Glycosides Tannin Carbohydrates Saponins Alkaloids Phytosterols	Antibacterial Antioxidant Cytotoxicity	[45]
			Ethanol	Flavonoids Tannins Alkaloids Carbohydrate Phenols Saponins Glycosides Phytosterols		
			Petroleum ether	Phenols Flavonoids Alkaloids		
			n-hexane	Phytosterols Alkaloids		
			Chloroform	Phenols Flavonoids Alkaloids		
			Water	Carbohydrate Phenols Flavonoids Tannin Saponins Glycosides Alkaloids		
5.	Bark	Microwave-assisted extraction	Water	Flavonoids Terpenoids	Antioxidant Antimicrobial	[46]
6.	Bark Leaf	Incubation	Ethanol	Phenolics Tannins Flavonoids Phytosterols	Antioxidant Antimicrobial	[47]
7.	Bark	Ultrasound-assisted extraction	Ethanol	Not mentioned	Antimicrobial	[48]
8.	Bark	Ultrasound-assisted extraction	Chloroform	Not mentioned	Determination of isoquinoline alkaloid	[49]
9.	Bark	Decoction	Water	Gallic acid Ellagic acid Luteolin	Cardiotonic property	[50]
10.	Bark	Soxhlet	Methanol	Tannin Ellagic acid	Anthelmintic	[51]
11.	Bark	Decoction	Water	Not mentioned	Ayurvedic formulation	[52]
12.	Bark	Hot continuous percolation-soxhlet	Ethyl alcohol	Not mentioned	Cardioprotective effect	[18]
13.	Bark	Percolation	Ethanolic	Flavonoids Tannins Glycosides Alkaloids Terpenoids	Hypoglycemic effect	[53]
14.	Bark	Hot continuous percolation-soxhlet	Water	Arjunolic acid Terminoic acid	Catecholamine-induced myocardial fibrosis and oxidative stress	[54]
15.	Bark	Hot continuous percolation	Water	Glycosides Flavonoids Polyphenols Saponins Terpenoids	Pulmonary hypertension	[55]
16.	Bark	Hot percolation	Methanol	Tannins and Phenolic Glycosides Flavonoids Polysterols Alkaloids Carbohydrate Proteins Triterpenoids Saponins	Osteogenic activity	[56]
17.	Bark	Cold percolation	Hydroalcoholic	Tannins Phenolics Sitosterol Anthraquinone Glycosides Alkaloids Flavonoids	Hypolipidemia activity	[57]
18.	Bark	Hot continuous percolation-soxhlet	Ethyl alcohol	Tannins Phenolics Sitosterol Anthraquinone glycosides Alkaloids Flavonoids	Atherogenic-induced dyslipidemia and metabolic syndrome	[58]
19.	Leaf	Maceration	Phosphate buffered saline	Terpenoids Flavonoids Saponins	Inhibition of T-cell antigen	[59]
20.	Bark	Cold maceration	Ethyl alcohol	Phytosterol β-sitosterol	Antioxidant potential of TA	[60]
21.	Bark	Reflux	Methanol	Gallic acid Catechin Chlorogenic acid Caffeic acid Ferulic acid p-coumaric acid absent in macerated Myricetin Quercetin Kaempferol	Cardioprotective Potential	[61]
		Maceration	Glycerin/Methanol			
22.	Bark	Maceration	Ethanol	Phenolic compounds	Anticancer activity of TA onHuman hepatoma cell (HepG2)	[62]
23.	Bark	Soxhlet	Butanol	Tannins and Phenolics Triterpenoids Saponins Anthraquinone glycosides Alkaloids Flavonoids	Cardioprotective potential in doxorubicin-induced cardiotoxicity	[63]
24.	Leaf	Maceration	Methanol	Alkaloids Triterpenoids Tannins Flavonoids	Analgesic and Anti-inflammatory activity	[17]
25.	Bark	Soxhlet	Methanol Ethanol Petroleum Ether Chloroform n-hexane	Flavonoids	Antioxidant	[64]

**Table 2:** Summary of green synthesized nanoparticles using TA extracts for various biomedical applications.

S. No	Name	Synthesis Method	Extract/Organism Used	Color of Nanoparticles Solution	Average Nanoparticle Size (nm)	Shape of Nanoparticles	Optimal Dosage	Application	Reference
1.	Silver	Incubation	Aqueous leaf	Yellow to dark brown	5–20	Spherical	Nil	Antimicrobial	[121]
2.	Silver	Hot plate magnetic stirring	Aqueous bark	Dark brown	65	Spherical	Nil	Antibacterial	[114]
3.	Silver	Shaking incubation	Endophytic bacteria from TA bark	Dark brown	42.2	Spherical	Nil	Antimicrobial	[113]
4.	Silver	Incubation	Aqueous fruit, bark, and foliage	Dark brown and intense brown	-	Spherical and irregular shape	Nil	Antimicrobial	[116]
5.	Silver	Incubation	Aqueous bark	Dark brown	34–70	Spherical	Nil	Cytotoxicity	[122]
6.	Silver	Microwave irradiation	Aqueous bark	Dark brown	10–15	Spherical	Nil	Antioxidant and antimicrobial	[123]
7.	Silver	Incubation	Aqueous bark	Dark brown	Non–uniform	Spherical and arbitrary	Nil	Antibacterial	[124]
8.	Silver	Incubation	Aqueous bark	Dark brown	20–50	Spherical	Nil	Larvicidal	[125]
9.	Silver	Microwave irradiation	Aqueous bark	Dark brown	20–30	-	Nil	Antimicrobial and anticancer	[126]
10.	Silver	Incubation	Endophytic fungi from TA leaves	Dark brown	45, 55	-	Nil	Antifungal	[127]
11.	Silver	Incubation	Aqueous bark	Dark brown	40–50	Spherical	Nil	Antibacterial	[128]
12.	Silver	Microwave irradiation	Aqueous and methanolic bark	Dark brown	20–50	Spherical	Nil	Antibacterial and antibiofilm	[129]
13.	Gold	Boiling	Aqueous bark	Dark brown	Nil	Nil	175 µg/kg/day	Nephrotoxicity	[110]
14.	Gold	Incubation at room temperature	Ethanolic arjunolic acid	Dark brown	185 nm	Spherical	Nil	Entrapment study and release study of gel-gold nanoparticle	[130]
15.	Gold	Boiling	Aqueous bark	Dark brown	7–20 nm	Spherical	175 µg/kg/day	Hepatotoxicity	[131]
16.	Gold	Incubation at room temperature	Aqueous leaf	Yellow to dark red	20–50 nm	Spherical	Nil	Mitotic cell division and pollen germination	[132]
17.	Gold	Drop wise addition	Aqueous bark	Violet to pinkish red	15–20 nm	Triangular, tetragonal, pentagonal, hexagonal, rod, and spherical	Nil	Catalysis	[119]
18.	Gold	Stirring	Aqueous bark	Pale yellow to ruby red	Nil	Nil	175 µg/kg/day	Reproductive dysfunction	[112]
19.	Gold	Stirring	Aqueous bark	Pale yellow to ruby red	20–40 nm	Spherical	175 µg/kg/day	Hematological alterations	[133]
20.	Gold	Boiling	Aqueous fruit	Yellow to reddish wine	25 nm	Spherical	Nil	Seed germination activity	[10]
21.	Gold	Incubation	Aqueous leaf	Light yellow to bright red	15–30 nm	Spherical	Nil	Antibacterial	[134]
22.	Gold	Stirring	Ethanolic bark	Yellow to pink and ruby red	3–70 nm	Spherical and triangular	50 µg/mL	Neuroprotective potential	[120]
23.	Copper	Shaking	Aqueous bark	Blue to brown	10–26	Spherical	Nil	Antimicrobial	[135]
24.	Copper	Microwave irradiation	Aqueous bark	Pale yellow to dark brown	23	Spherical	Nil	Antimicrobial and antioxidant	[46]
25.	Copper	Microwave irradiation	Aqueous bark	Light yellow to black	20–30	Nil	Nil	Antimicrobial and anticancer	[126]
26.	Iron	Microwave irradiation	Aqueous bark	Yellow to greenish black	20–80 nm	Globular	Nil	Photo degradation	[136]
27.	Palladium	Incubation	Aqueous bark	Dark brown	4–16	Spherical	Nil	Catalysis	[137]
28.	Zinc	sonication, wet chemical, and hydrothermal	Aqueous bark	Nil	43, 34, 21	Spherical	25 mg mL^−1^	Toxicity and antibacterial	[111]

## Data Availability

All the data and materials that support the results or analyses presented in their paper are freely available upon request.
